# Cofilin-1 and phosphoglycerate kinase 1 as promising indicators for glioma radiosensibility and prognosis

**DOI:** 10.18632/oncotarget.19025

**Published:** 2017-07-05

**Authors:** Wenbo Sun, Hua Yan, Chunfa Qian, Chenhan Wang, Mengjie Zhao, Yuchi Liu, Yujie Zhong, Hongyi Liu, Hong Xiao

**Affiliations:** ^1^ Department of Neurosurgery, Nanjing Medical University Affiliated Brain Hospital, Nanjing, China; ^2^ Department of Neuro-Psychiatric Institute, Nanjing Medical University Affiliated Brain Hospital, Nanjing, China

**Keywords:** CFL1, PGK1, glioma, radiosensibility, prognosis

## Abstract

Glioma is a primary malignancy in central nervous system. Radiotherapy has been used as one of the standard treatments for glioma for decades. Since radioresistance can reduce the curative efficacy of radiotherapy in glioma, investigating the cause of radioresistance and predicting the tumour radiosensibility appeared particularly important. We previously reported that CFL1 and PGK1 are over-expressed in radioresistant U251 glioma cells. In this study, the level of CFL1 and PGK1 of 113 glioma tissues were measured by ELISA method. The relevance of the expression of these two proteins to radiosensibility was analyzed by mean test and multivariate logistic regression. The survival analysis was carried out in 85 irradiated patients and 105 followed-up patients respectively. The relationship between protein expression and clinical parameters was explored in overall 113 patients, and the correlation between CFL1 and PGK1 were determined as well. Our results showed that the expression of CFL1 and PGK1 were significantly higher (*P* < 0.001) in radioresistant patients than others. The multivariate Logistic regression demonstrated that the expression of CFL1 (*p* < 0.001) and PGK1 (*p* < 0.001) were associated with radioresistance in glioma. The multivariate Cox regression in overall survival suggested that CFL1 level or PGK1 level could be the independent prognosis factor for poor prognosis in 113 glioma patients. In addition, CFL1 expression was positively correlated with PGK1 expression in glioma. The results suggested that as promising indicators, CFL1 and PGK1 could be used to evaluate glioma radiosensibility and prognosis. These two proteins could also be the potential therapeutic targets of glioma.

## INTRODUCTION

Glioma is the most prevalent and aggressive tumor in central nervous system [1]. Its major biological characteristics, including anomalous formation, widespread angiogenesis and infiltrative growth, make it rarely curable. The prognosis for patients with high-grade gliomas is generally poor [2, 3]. Radiotherapy has been the primary post-surgery adjuvant treatment for decades [4], however, the radioresistance reduces the curative efficacy of glioma radiotherapy.

Phosphoglycerate kinase 1 (PGK1) is a major enzyme used in the first ATP-generating step of the glycolytic pathway. It catalyzes the reversible transfer of a phosphate group from 1, 3-diphosphoglycerate to adenosine diphosphate (ADP) producing 3-phosphoglycerate and adenosine triphosphate (ATP) [5, 6]. It has been also revealed in the functions associated with up-regulating various malignances [7] such as inhibiting tumour vascularity in tumorigenesis [8] and regulating DNA replication and repair [9].

Cofilin-1(CFL1) belongs to actin-depolymerizing/cofilin superfamily. The CFL1 gene is widely distributed in the non-muscle tissues [10]. CFL1 can bind to actin directly to accelerate the depolymerization at the end of the actin filaments and expedite the actin filaments turn over [11, 12]. Therefore, it plays an essential role to control actin dynamics, which is important for tumor cell proliferation and migration. CFL1 expression is critically associated with cell migration and tumor invasion [13], and can be a marker of tumor progression [14].

Current treatment of gliomas is surgery combined with chemotherapy and/or radiotherapy. However, many tumors show a high resistance to these interventions, and recurrences are frequent. Radiotherapy has been used for a long time. The dose-effect of radiotherapy and radiation damage to the central nervous system have been studied since 1980 [15, 16], but the comprehensive responding of cells and the mechanism of tumor cell radioresistance are still not been illuminated [17]. Individual patients respond to radiotherapy and suffer radiation damage differently because their tumor cells have distinct cellular radiosensibility [18, 19]. Until now, there is no biomarker for tumor cell radiosensibility recognized. In this study, we want to find such biomarker(s) to indicate patient's tumor cell radiosensibility prior to the radiotherapy; therefore to limit the tumor cell radioresistance and increase the radiotherapy efficacy.

By using 2D-HPLC-MS/MS method, we found that both PGK1 and CFL1 are abundantly expressed in the radioresistant glioma tissue from patients [20]. We also found that the over-expression of CFL1 or PGK1 can reduce glioma radiosensibility *in vivo* and *in vitro*, respectively [21–24]. In this study, we investigated the association between protein expression and radiosensibility in clinical glioma patients at first. Subsequently, the correlation of the expression of CFL1 and PGK1 with clinical characteristics was determined using the survival analysis in order to validate the prognosis of post-radiotherapy and overall glioma patients respectively.

## RESULTS

### The follow-up information and the association between protein expression and clinicopathologic parameters

The characteristics of a total of 113 patients are comprised 63 male and 50 female, the rank of age which were from 7 to 73 years old and mean age was 52.29 ± 14.17 years. The patient of tumor total resection was 94, and tumor partial resection was 19. There are 84 and 28 patients respectively in incipience and recurrence. Eighty five patients were received radiotherapy and 20 were not. Sixty five patients were received chemoradiotherapy and others were not. There are 29 patients classified as WHO grade II, 35 patients classified as WHO grade III and 48 patients classified as WHO grade IV (Table 1). The medial follow-up time was 17 months. There were 28 patients got disease progression/recurrence after the surgery. During the study period, a total of 94 glioma patients died.

**Table 1:** Clinical characteristics of glioma patients enrolled in this study

	n	CFL1 expression		PGK1 expression	
		Mean ± SD(Pg/g)	*P*	Mean ± SD(Pg/g)	*P*
Overall	113	408.43 ± 312.70	-	309.55 ± 236.75	-
Gender			0.991		0.960
Male	63	408.74 ± 299.33		308.56 ± 216.25	
female	50	408.04 ± 331.85		310.80 ± 262.57	
Age, 52.29 ± 14.17(years)	113	-	0.071	-	0.077
WHO grade			0.005*		0.005*
II	29	302.60 ± 205.70		223.33 ± 140.63	
III	35	400.12 ± 325.32		314.90 ± 283.72	
IV	49	477.01 ± 341.95		356.76 ± 235.45	
Preoperative situation			0.105		0.236
Incipience	84	427.60 ± 333.46		316.79 ± 255.34	
Recurrence	28	352.91 ± 239.17		288.59 ± 174.12	
Extent of surgery			-		-
Total removal	94	429.28 ± 317.58		330.08 ± 244.15	
Residual	19	305.29 ± 271.69		207.99 ± 166.38	
Postoperative radiotherapy	85	433.35 ± 336.92	-	331.56 ± 254.41	-
Postoperative chemotherapy	65	414.20 ± 317.02	-	321.94 ± 259.14	-

*Statistically significant.

The CFL1 and PGK1 expressions and its correlation with the clinical characteristics were shown in Table 1. The WHO grade were significantly positive correlated (*P* = 0.005) to the expressions of CFL1 and PGK1, and the gender, the age, incipience/recurrence were not (Table 1). The median expression of CFL1 was 316.13 pg/g and median expression of PGK1 was 225.53 pg/g. These two median expressions were chosen as the cut-off points of over/low expression groups.

### The association between protein expression and radiosensibility

### CFL1 and PGK1 expressions were significantly higher in radioresistant patients

**Figure 1:**
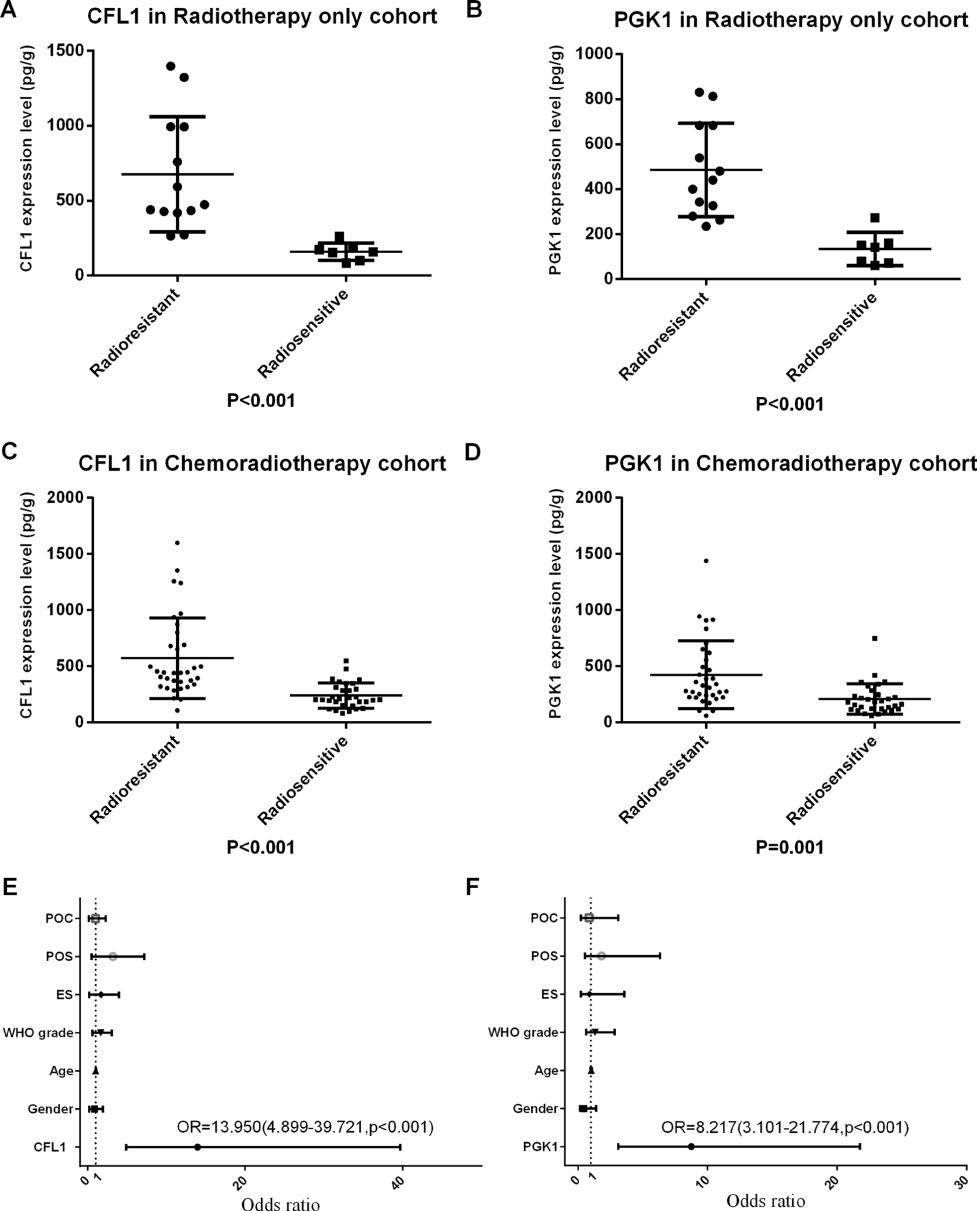
POC: postoperative chemotherapy; POS: preoperative situation; ES: extent of surgery; CFL1 and PGK1 expression evaluated the radiosensibility (**A**) CFL1 in RTO (*t*-test, *p* < 0.001); (**B**) PGK1 in RTO (*t*-test, *p* < 0.001); (**C**) CFL1 in CRT (Mann-Whitney *u*-test, *p* < 0.001); (**D**) PGK1 in CRT (Mann-Whitney *u*-test, *p* < 0.001); (**E**) Multivariate Logistic regression(enter method) has been performed with gender, age, WHO grade, extent of surgery, incipience/recurrence, chemotherapy and CFL1 expression, only CFL1 expression was statistics significant; (**F**) Multivariate Logistic regression(enter method) has been performed with gender, age, WHO grade, extent of surgery, incipience/recurrence, chemotherapy and PGK1 expression, only PGK1 expression was statistics significant.

Radiotherapy only cohort (RTO) was patients which were merely accepted postoperative radiotherapy (*n* = 20) and chemoradiotherapy cohort (CRT) which was patients simultaneously accepted postoperative chemo- and radiotherapy (*n* = 65). The comparisons of the CFL and PGK1 expression levels were shown significant differences (*P* < 0.001) between radiosensitive and radioresistant groups in both RTO cohort and CRT cohort (Figure 1A–1D). The results demonstrated that over-expression of CFL1 and PGK1 were associated with the radioresistance whatever the patients undergo the chemotherapy or not.

### CFL1 and PGK1 were associated with radiosensibility

Radiotherapy cohort was patients who have accepted postoperative radiotherapy (*n* = 85). The multivariate Logistic regression was performed with enter method to analyze the OR of the CFL1 and PGK1 expressions and clinicopathology parameters which were involved in gender, age, WHO grade, extent of surgery, preoperative situation and postoperative chemotherapy. The outcome revealed only protein expressions were the influential variables to radiosensibility. CFL1 expression Odds Ratio = 13.950 (95% CI = 4.899–39.721, *p* < 0.001), PGK1 expression OR = 8.217 (95% CI = 3.101–21.774, *p* < 0.001). (Figure 1E, 1F).

### The association between protein expression and the prognosis of glioma radiotherapy

### The over-expression of CFL1 and PGK1 were associated with poor prognosis of radiotherapy patients

**Figure 2:**
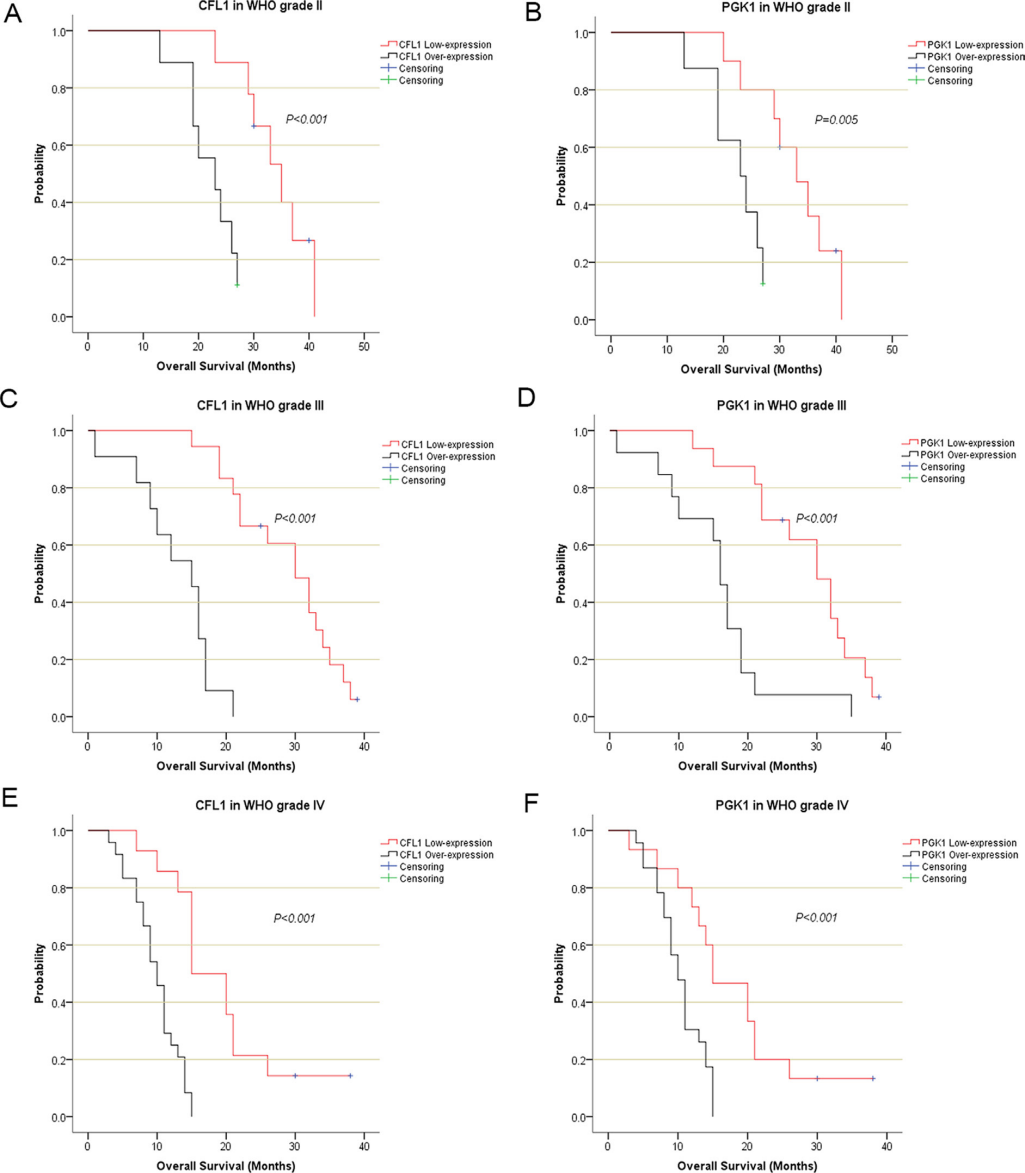
The Kaplan-Meier survival analysis described the survival curve of irradiated patients CFL1 and PGK1 expressions in each WHO grade . (**A**) CFL1 in WHO II; (**B**) PGK1 in WHO II; (**C**) CFL1 in WHO III; (**D**) PGK1 in WHO III; (**E**) CFL1 in WHO IV; (**F**) PGK1 in WHO IV.

Radiotherapy cohort was enrolled in this section. The Kaplan-Meier survival analysis was used to describe the survival curves of the CFL1 and PGK1 over-expression groups and low-expression groups within each WHO grade II, WHO grade III and WHO grade IV gliomas. Log-rank test was performed to verify the difference between the two groups. The results revealed there was a significant difference between the CFL1 and PGK1 over-expression group and low-expression group in each WHO grades (*p* < 0.001, Figure 2A–2F).

**Figure 3:**
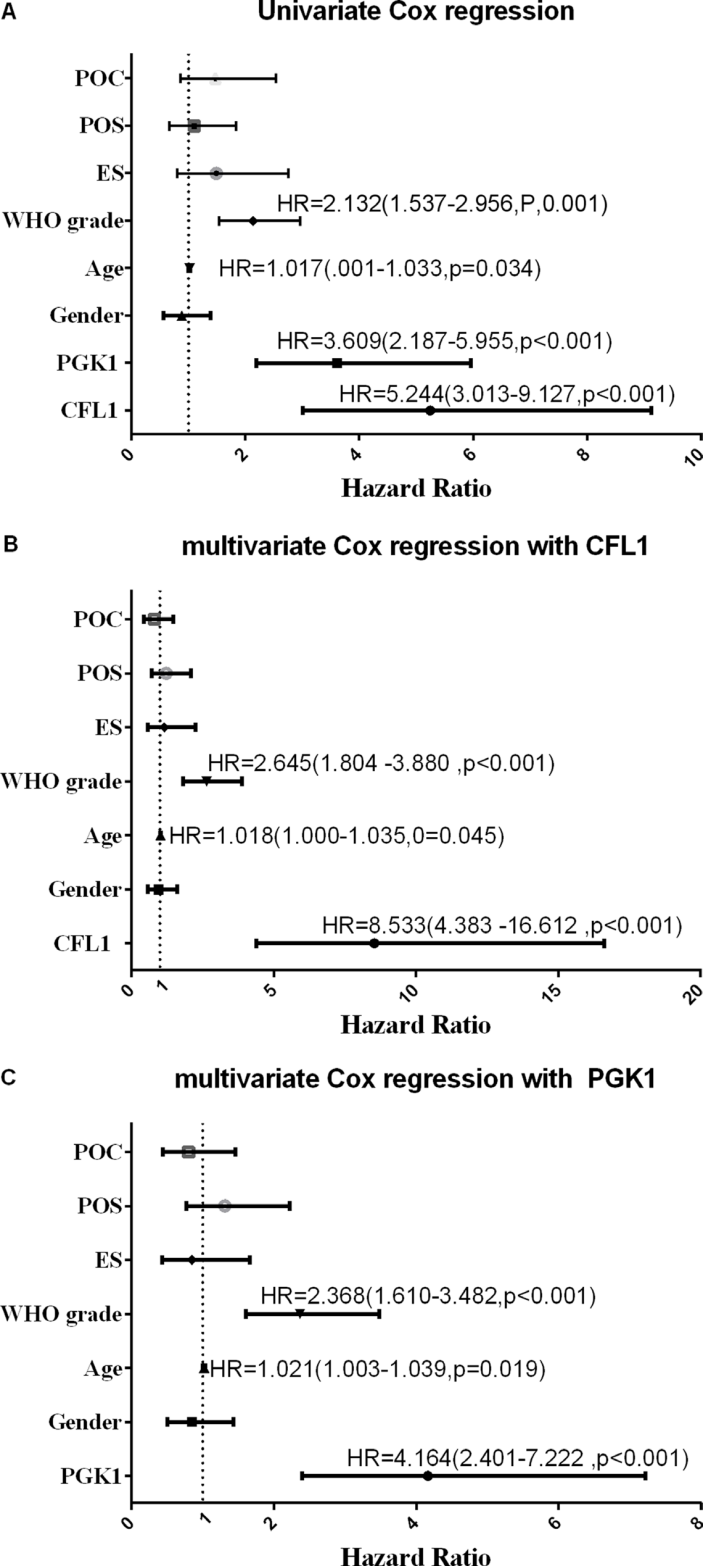
POC: postoperative chemotherapy; POS: preoperative situation; ES: extent of surgery; Univariate/multivariate Cox regression were performed to verify the predicting factors in irradiated patients (**A**) univrate Cox regression with all variables; (**B**) clinical parameters with CFL1 expression; (**C**) clinical parameters with PGK1 expression. No label variables was not significant (*p* > 0.05).

The outcome of multivariate Cox regression revealed that among Overall Survival (OS) Hazard Ratio (HR) of CFL1 over-expression group vs. low-expression group was 8.533 (*P* < 0.001) and HR of PGK1 over-expression group vs. low-expression group was 4.164 (*P* < 0.001) (Figure 3). Combined the outcomes of univariate and multivariate Cox regression, the CFL1 and PGK1 expression, WHO grade and age were the independent prognostic factors of OS in irradiated patients (Figure 3).

### Relationship between protein expression and prognosis of glioma

### The CFL1 and PGK1 over-expression were associated with the poor prognosis of glioma patients

**Figure 4:**
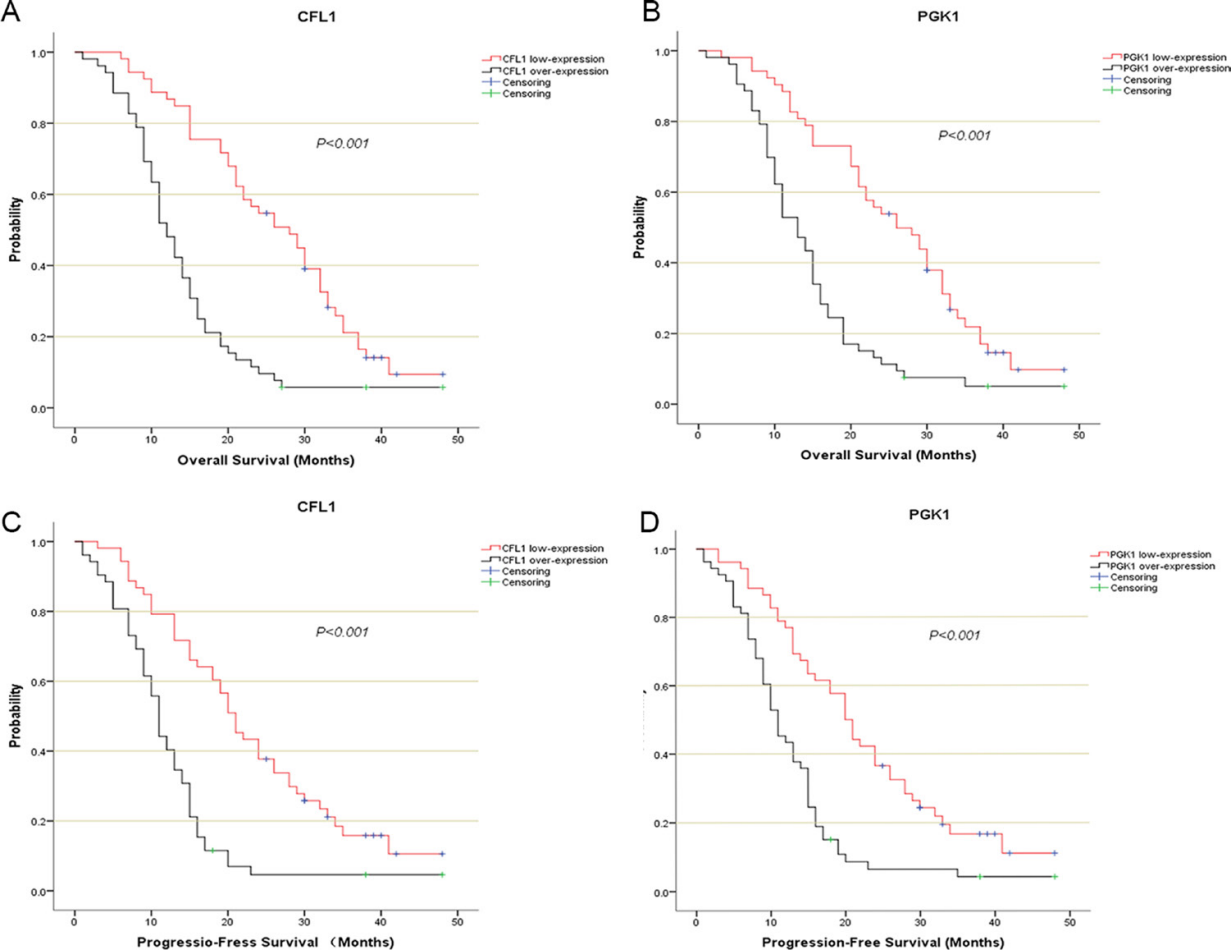
The kaplan-Meier survival analysis described the survival curve of CFL1/PGK1 over-expression and low-expression (**A**) CFL1 in OS of entire patients; (**B**) PGK1 in OS of entire patients; (**C**) CFL1 in PFS of entire patients; (**D**) PGK1 in PFS of entire patients. The Log-Rank test were performed to compare the difference of survival rate between each groups.

**Figure 5:**
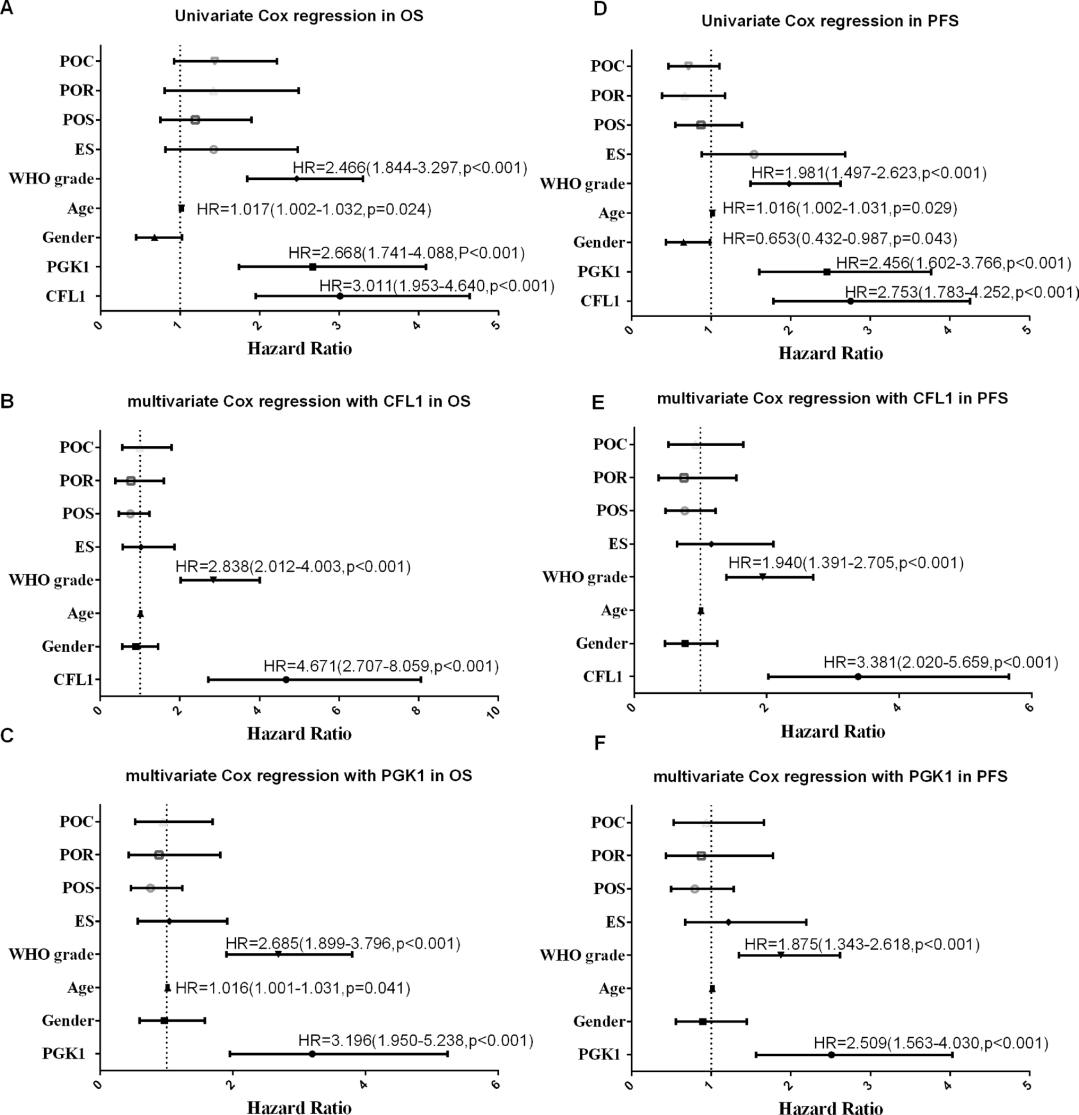
POC: postoperative chemotherapy; POR: postopeartive radiotherapy; POS: preoperative situation; ES: extent of surgery; univariate/multivariate Cox regreesion were carrird out to verify the independent prognosis factors in glioma patients of OS and PFS (**A**) univariate in OS; (**B**) multivariate with CFL1 in OS; (**C**) multivariate with PGK1 in OS; (**D**) univariate in PFS; (**E**) multivariate with CFL1 in PFS; (**F**) multivariate with PGK1 in PFS. No label variables were not significant (*P* > 0.05).

One hundred and five glioma patients who had been followed-up were enrolled in this analysis. The Kaplan–Meier survival analysis and log-rank test suggested that over-expression group showed poorer prognosis than low-expression (Figure 4A, 4B). The univariate/multivariate Cox proportional hazards model revealed The CFL1 and PGK1 over-expression were the independent prognosis factors of glioma patients. Multivariate Cox regression indicated that the adjusted HR of CFL1 over-expression group vs. low-expression group was 4.671 (2.707–8.059, *P* < 0.001) and HR of PGK1 over-expression group vs. low-expression group was 3.196 (1.950–5.238, *P* < 0.001) (Figure 5A–5C).

### The CFL1 and PGK1 over-expression were associated with glioma progression

The survival curves showed there were a significant difference between over-expression and low-expression groups of two proteins (Figure 4C, 4D). In univariate and multivariate Cox regression, the results demonstrated The CFL1 (HR = 3.381, 95% CI = 2.020–5.659, *p* < 0.001) and PGK1 (HR = 2.509, 95% CI = 1.563–4.030, *p* < 0.001) over-expression were associated with the glioma progression (Figure 5D–5F).

### The correlation between CFL1 and PGK1

**Figure 6:**
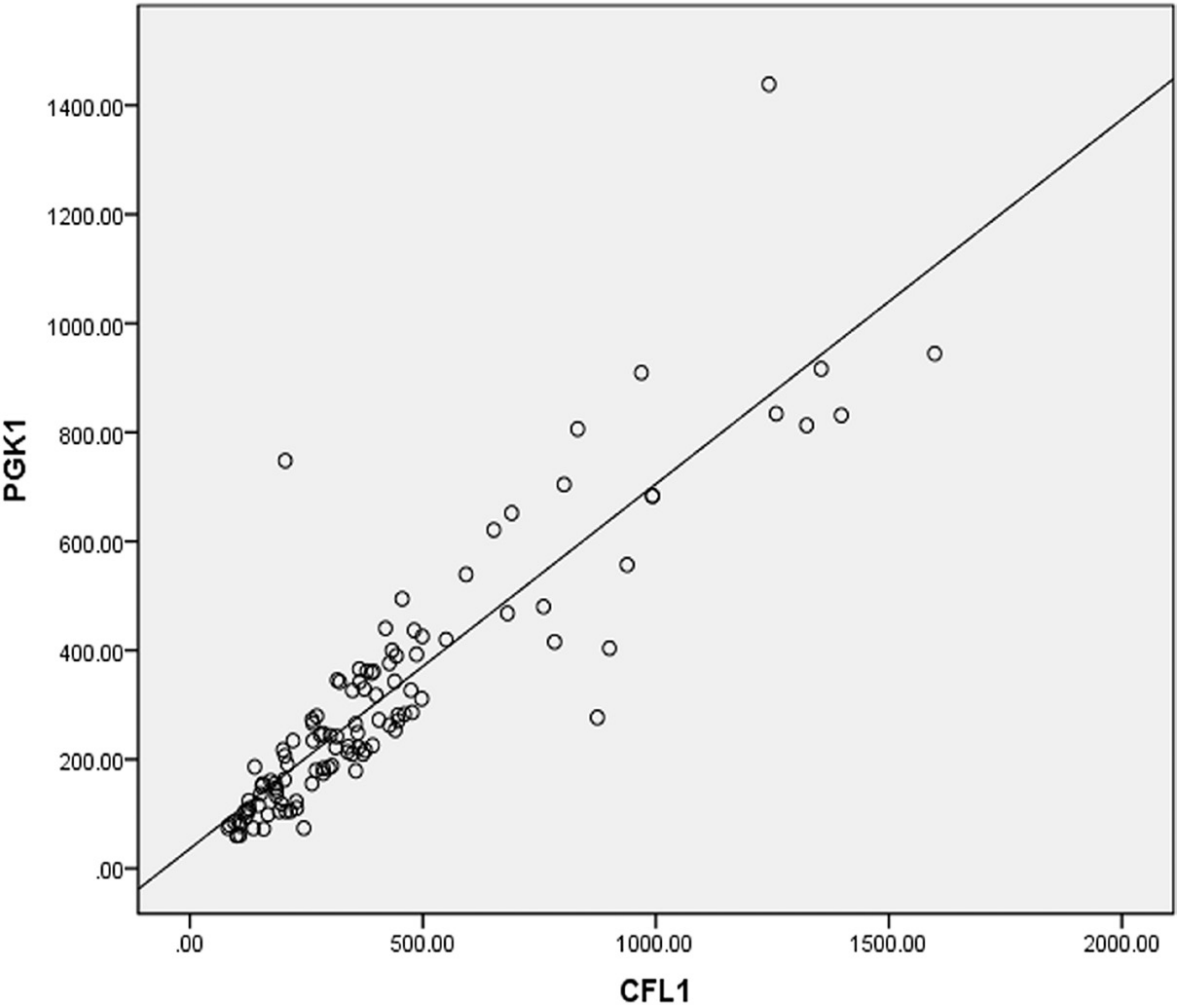
The pearsion coefficient (r) between CFL1 and PGK1 expression was 0.884, *p* < 0.001

The correlation analysis was performed on a total of 113 patients. The notably positive correlation between CFL1 and PGK1 was shown in Figure 6. (*r* = 0.884, *P* < 0.001).

## DISCUSSION

In summary, we investigated the relevance between both CFL1 and PGK1 protein expressions and glioma tumor radiosensibility. We found that CFL1 and PGK1 expressed more in the radioresistant patients no matter the chemotherapy used or not. Also CFL1 and PGK1 over-expression are significantly associated with the radioresistance patients in multivariate regression model.

The postoperative radiotherapy is an established standard treatment for glioma patients. In general, success or failure of this standard clinical radiation treatment is determined by the 4 R's of radiobiology: repair of DNA damage, redistribution of cells in the cell cycle, repopulation, and reoxygenation of hypoxic tumor areas. In addition to these traditional four Rs, the cellular radiosensibility, including both intrinsic and extrinsic radiosensitivity, has to be considered as an essential factor for radiotherapy too [25, 26]. Having an individualized radiotherapy plan based on each patient's radiosensibility is necessary for increasing the radiation treatment efficacy [17]. Thus, the radiosensibility biomarker(s) can be very useful in glioma radiotherapy.

PGK1 is one of the glycolytic enzymes [6]. PGK1 over-expression has been shown to correlate with the tumorigenesis in pancreatic ductal adenocarcinoma, liver cancer and breast cancer [27–29]. It has been also reported that PGK1 stimulates the metastasis in prostate cancer [30], and promotes the tumor invasion and metastasis in gastric cancer [31]. CFL1, on the other hand, binds to actin to depolymerize the actin microfilament [12], so that it plays a significant role in breast tumour migration and metastasis [32]. Dysregulation of CFL1 may impact tumor proliferation and invasion [13, 33]. In vulvar tumor, CFL1 has been reported to have an effect on carcinogenesis and progression [14]. Thus, both PGK1 and CFL1 have some effects on tumor invasion, proliferation and metastasis, but whether they are associated with tumor cell radiosensibility remains unknown.

PGK1 can be up-regulated by hypoxia-inducible factor 1 (HIF-1), the product of hypoxia [34], suggesting that PGK1 activity may be related to the hypoxia. In general, tumors develop region of hypoxia [34], therefore the PGK1 activity in the cancer cells is higher than in the normal cells. Under hypoxia, the photon generated by ionizing radiation (IR) arouses less oxygen radicals causing tumor cell radioresistance [17]. It is possible that over-expressed PGK1 is one of the extrinsic factors of tumor cell radiosensibility [25, 35]. As a glycolytic enzyme, PGK1 can provide energy to tumour cells and increase tumour migration, invasion and cell viability [19, 21]. In addition, PGK1 can decrease the secretion of angiostatin as a disulphide reductase and promote tumor angiogenesis [8]. It can also play a role in the modulation of DNA repair [9], a process directly correlated to the tumor cell radioresistance [36]. PGK1 over-expression has been shown to associate with the poor prognosis in breast cancer, liver cancer, lung cancer, gallbladder cancer and prostate cancer [28, 29, 32, 37–39]. PGK1 can also affect gastric cancer progression [40].

CFL1 has been suggested to block tumor cell apoptosis [41], which is beneficial to tumor cell growth and decreases tumor cell radiosensibility [40]. Our pervious study demonstrated that CFL1 over-expression can significantly decrease U-251 cell cycle arrest of G2 phase [22], indicating that CFL1 can accelerate DNA repair after the ionizing radiation [42]. It was also reported that CFL1, as a downstream target of Rho-associated coiled-coil kinase (ROCK), plays a key role in glioma progression [43].

We have reported that both PGK1 and CFL1 are over-expressed in radioresistant U-251 cell, and the over-expression of PGK1 and CFL1 are corresponded with the enhancement of cell migration, invasion and viability [21, 22]. In this clinical research, we further demonstrated that both CFL1 and PGK1 are also over-expressed in radioresistant glioma patients. Collectively our results indicate that over-expression of PGK1 and CFL1 can be closely related to glioma radiosensibility.

Glioma has a high mortality rate and the prognosis for patients with high-grade gliomas is usually poor [44]. Although pathologic evaluation of the tumor is used to determine the glioma grade, having prognostic biomarkers to assess glioma has been considered better [45]. Here we show that both The CFL1 and PGK1 expression can be used as prognosis factors for glioma patients (Figure 4), because both The CFL1 and PGK1 over-expression were associated with poor prognosis of radiotherapy cohort and entire followed-up cohort.

In this study, both CFL1 and PGK1, as influential factors of radiosensibility, were expressed significantly higher in the radioresistant glioma patients. The over-expression of CFL1 and PGK1 were also associated with the poor prognosis of glioma patients. In addition, CFL1 expression was positively correlated with PGK1 expression. Thus, both CFL1 and PGK1 can be used as promising biomarkers for radiosensibility, prognosis and progression in glioma; and be potential therapeutic targets of glioma as well. An interesting highlight in our study is the positive correlation between the expression of CFL1 and PGK1, suggesting a potential molecular reaction mechanism between CFL1 and PGK1. The further investigation of such mutual relationship between these proteins and tumor cell radiosensibility is necessary.

## MATERIALS AND METHODS

### Patients

The glioma tissues were obtained from 113 patients who underwent the surgery of cerebral glioma at the Department of Neuron Surgery in Nanjing Medical University Affiliated Brain Hospital, from May 2012 to October 2015. The patients recruited in this study at least follow-up for 2 years or have died. The research protocol was approved by the Ethical Committee of Nanjing Medical University, Affiliated Nanjing Brain Hospital (Permit No: 2011-KY001). At the end of the follow-up, there were 7 patients are missed follow-up. Radioresistant group was composed of irradiated patients with (i) WHO grade II/III and OS within 24 months; (ii) WHO grade IV and OS within 14 months. The rest of the irradiated patients were divided into radiosensitive group.

### Tumor tissues CFL1 and PGK1 determination

The tissues were collected after the rapid pathologic diagnosis and transferred, using liquid nitrogen, into the −80°C until analysis. For measurement of The CFL1 and PGK1 over-expression tissues total protein was extracted at first. Then CFL1 and PGK1 in glioma were detected by ELISA kit (RB Scientific, UK). The detection wavelength was at 450 nm.

### Statistical analysis

All the statistical analysis was performed in Statistical Package for the Social Sciences 22.0 (IBM Corporation, Armonk, NY) and Statistical Analysis System 9.3 (Sas Institute INC, North Carolina). The association between clinical characteristics and protein expression was assessed by Pearson test, Spearman test or *t*-test. The difference analysis in RTO cohort and CRT cohort carried out the *t*-test or Mann-Whitney *U* test when appropriate. Multivariate logistic regression was performed with enter method to validate the influential variables of radiosensibility. The Kaplan–Meier survival analysis was used to describe the survival curve. The log-rank test was evaluated in the difference between survival curves. The univariate and multivariate Cox proportional hazards model with enter method were used to estimate the Hazard Ratio (HR) and 95% confident interval (CI) for death risk. The cut-off points were the expression of CFL1 and PGK1 that from median to maximum and the highest-HR cut-off point has been chosen for further analysis. Pearson test was performed to assess the correlation between CFL1 and PGK1. Overall Survival was defined as the survival time from surgery to death or the date to the last follow-up. Progression-Free Survival was defined as the survival time from surgery to glioma recurrence or death, or the date to the last follow-up. A two-tailed *p* < 0.05 was regarded as statistical significance.

## Abbreviations

CFL1: cofilin-1
PGK1: phosphoglycerate kinase 1
OS: overall survival
PFS: progression-free survival
RTO: radiotherapy only cohort
CRP: chemoradiotherapy cohort
HR: hazard ratio
CI: confident interval
